# Correlation of ultrasonography and surgical outcome in patients with testicular torsion

**DOI:** 10.11604/pamj.2020.36.45.21824

**Published:** 2020-05-29

**Authors:** Ismail Abdul Sattar Burud, Syed Mahmud Irfan Alsagoff, Roshinipriya Ganesin, Sumitta Thamil Selvam, Nor Aniza Binti Zakaria, Mahadevan Deva Tata

**Affiliations:** 1 Department of Surgery, International Medical University, Clinical campus, Seremban, Malaysia; 2 Undergraduate student International Medical University, Clinical Campus, Seremban, Malaysia; 3 Department of Radiology Hospital Tuanku Ja’afar, Seremban, Malaysia; 4 Department of Surgery Hospital Tuanku Ja’afar, Seremban, Malaysia

**Keywords:** Testicular torsion, testicular pain, orchidectomy, ultrasonography

## Abstract

**Introduction:**

Testicular torsion is a surgical emergency that is caused by twisting of the spermatic cord and its content. This condition causes irreversible changes after 6 hours. Early recognition and management of testicular torsion is important for testicular salvage and preservation of fertility.

**Methods:**

This is a retrospective study done on all patients who presented with acute scrotal pain from January 2013 to December 2017. The data collected included the patient's age, symptoms, the time duration between the onset, ultrasound, and surgery, ultrasound findings with Doppler and the surgical intervention. Statistical analysis was performed using SPSS 25.0. Data are presented as mean (SD) values. Differences between groups and predictive values were calculated using Chi-square, t-test and Mann-Whitney U-test and are expressed by value with 95% CI.

**Results:**

The total number of patients who presented with acute scrotal pain were 88. Testicular torsion was diagnosed in 55 (62.50%) of the patients, 17 (19.32%) had epididymis-orchitis, 5 (5.68%) had torsion of appendage/cyst, and 11 (12.50%) had normal testis. Ultrasound has a sensitivity and specificity of 88.24% and 68.40% respectively. It is a good tool to detect testicular torsion but it is operator dependent. Positive predictive value was 83.33% and negative predictive value was 76.47%. When ultrasound is combined with clinical findings the rate of negative exploration is reduced by 10%.

**Conclusion:**

Good medical history, appropriate clinical evaluation and performing an ultrasound of the scrotum are important in testicular torsion. US evaluation in cases presented after 24 hours does not change the outcome.

## Introduction

Testicular torsion (TT) is a surgical emergency caused by the twisting of the spermatic cord and its contents. The presentation is acute scrotal pain and early diagnosis is important to salvage the testis. Patients with scrotal pain are often seen by general practitioners and other specialists mainly surgeons and urologists. Acute scrotal pain is also caused by other conditions like torsion of testicular appendages, Epididymo-orchitis (EO), inguinal hernia, hydrocele, trauma, testicular tumours, varicocele. Testicular torsion, epididymo-orchitis, and torsion of the Testicular Appendix (TA) are the three most common causes of 'acute scrotum' in children [1, 2]. The most important aspect that treating doctors should be aware of is time. The need for early treatment of spermatic torsion to avoid testicular infarction is well recognized [3, 4]. Spermatic cord torsion reduces blood supply to the testis, which subsequently leads to haemorrhage, infarction, and necrosis. Many studies have shown that testicular infarction begins within the first 2 hours of spermatic cord torsion onset, irreversible damage occurs after 6 hours, complete infarction develops after 24 hours [5].

The annual incidence of TT is 3.8% in males aged <18 years [6]. It has a bimodal distribution, with peaks in the perinatal period and in adolescence, which reflects the clinical distinction between extravaginal torsion in new-borns and intravaginal torsion in older children [7]. History and clinical examination are important in diagnosing torsion testis and differentiating from other causes of acute scrotal pain. Short pain duration, nausea or vomiting, high position of the testicle, abnormal ipsilateral cremasteric reflex and scrotal skin changes have been identified, mostly in retrospective studies, as being associated with an increased likelihood of TT [7, 8]. Treating doctors resort to various investigatory test which includes urinalysis and color Doppler ultrasonography. Doppler ultrasonography has a high sensitivity (88.9%) and specificity (98.8%) preoperative diagnostic tool with a 1% false-negative rate [9]. Imaging studies such as MRI is a very accurate tool, providing sensitivity and specificity of 93% and 100%, respectively [10]. However, it is very expensive and time-consuming, which will delay the treatment of TT.

## Methods

This is a retrospective study that is done on all patients who presented with acute scrotal pain throughout the five years period which started from January 2013 to December 2017. The data was collected from Hospital Tuanku Ja´afar, Malaysia with the institutional review board and ethical approval that was obtained before beginning the study. The data collected regarding the patient´s age, symptoms, the time between the onset, ultrasound, and surgery, ultrasound findings with Doppler findings and the surgical outcome of the patients. A centralized electronic medical record system was used to ensure that all hospital visits, imaging studies, and patients follow-up were included.

The Ultrasound (US) was performed during office hours by experienced radiologists. The determination of the sonomorphology of the scrotum and epididymis, including echogenicity and echotexture, was done. The echogenicity was then described as, normal echogenicity (homogeneous pattern) and diffuse or focal hyper- or hypoechogenicity (a heterogeneous and homogeneous pattern). The evaluation included measuring the bilateral testicular size and volume. Finally, central and peripheral perfusion of the testicle with colour Doppler US in comparison with the other side was assessed. Ultrasound diagnosis of TT was made if there was a reduce/absent perfusion or an abnormal echogenicity. Based on these clinical predictions and radiological findings, the eventual prediction was made before the surgical exploration took place. Statistical analysis was performed using SPSS 25.0. Data are presented as mean (SD) values. Differences between groups and predictive values were calculated using Chi-square, t-test and Mann-Whitney U-test and are expressed by value with 95% CI. p value less than 0.05 were considered statistically significant.

## Results

**Figure 1:**
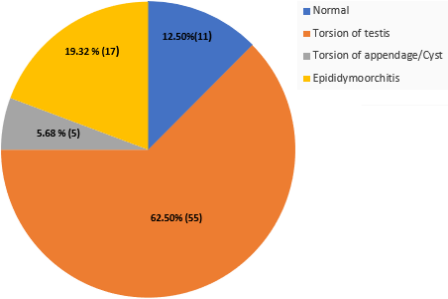
Diagnosis of patients presenting with acute scrotal pain

During the five-year study, 88 patients presented with acute scrotal pain. The diagnosis are as shown in Figure 1. Ultrasound was performed in 66 patients, however, only 53 patients had the complete results. Predictive values were analyzed using Chi-square, t-test and Mann-Whitney U-test when appropriate using SPSS ver. 25.0. The median age of the study population was 17 years (5-68 years). Analysis using Mann-Whitney U-test showed no significant difference in mean age between those with or without TT the mean (SD) age of boys with TT was 18.11 (6.83) years (P > 0.05). Besides, swelling (OR: 2.83, 95% CI) has a positive predictive value for TT.

Based on clinical judgment alone the negative exploration rate was 45.5%. However, on combining both clinical findings and ultrasound, a negative exploration rate was reduced by about 10%. The analysis of clinical features is shown in Table 1. Out of 55 patients diagnosed with TT, 33(65.5%) required orchidectomy due to non-viable testis at exploration. The median range of the patient who had orchidectomy was 16 (5-33). With the t-test, there was a significant difference (p=0.046) between the mean (SD) of the patient who had orchidectomy and orchidopexy which was 16.78 (5.986) and 20.63 (7.747) respectively. Table 2 shows the duration of pain and the outcome of the surgery for testicular torsion. The duration of pain was not a significant factor in determining TT (P=0.356). However, orchidectomy was more common in patients with pain duration >24 hours (20/30, 66.7%) and pain duration within 6-24 hours (13/14, 92.9%).

**Table 1:** Symptoms and clinical findings in presenting patients

Features	TT group, N= 55 N (%)	Non-TT group, N=33 N (%)	Chi-square ( P-value)
Fever	4 (7.27)	5 (15.15)	0.238
Nausea	4 (7.27)	1 (3.03)	0.405
Vomiting	7 (12.72)	2 (6.06)	0.318
Dysuria	3 (5.45)	6 (18.18)	0.56
Swelling	40 (72.73)	16 (48.45)	0.022
Previous Orchidectomy/Orchidopexy	1 (1.8)	0 (0)	0.436

**Table 2:** Duration of pain and outcome of surgery

Pain Duration (Hours)	>24, N= 30 N (%)	6-24 N= 14 N (%)	<6 N= 11 N (%)	Chi-square (P-Value)
Orchidopexy	10 (33.3)	1 (7.1)	8 (72.7)	0.003
Orchidectomy	20 (66.7)	13 (92.9)	3 (27.3)	

Ultrasound evaluation of TT shows sensitivity and specificity of 88.24% and 68.40% respectively. The positive predictive value was 83.33% and negative predictive value (NPV) was 76.47%. Doppler ultrasound resulted in false-positive findings for 6 patients (31.6%) and false-negative finding for 4 patients (11.8%). Table 3 shows the sonographic features of the study population. Echogenicity on ultrasound shows that heteroechogenicity was more often seen in TT rather than in patients diagnosed with other pathologies (16/34 vs 4/19, P=0.003). Furthermore, hypoechogenicity is also shown to be more common in TT than patients without TT (9/34 vs 1/19, P=0.003). Doppler US showed reduced perfusion of the affected testis to be more associated in the TT group (27/34 vs 4/19, P=<0.001). The size of testis on ultrasound had a significant difference in TT and non-TT group (P=0.024). TT is more common in patients with either increase (n=15, 71.4%) or decrease (n=13, 81.4%) in the testicular size compared to other pathologies. The analysis of sonographic features of patients with TT who underwent orchidectomy and orchidopexy are shown in Table 4. only reduction in perfusion was significant findings (P value 0.002, OR: 14.46, 95% CI) shows a positive predictive value for TT.

**Table 3:** Sonographic findings in patients presenting with TT and without TT

Features	Orchidopexy, N= 6 N(%)	Orchidectomy, N=28 N(%)	Chi-square (P-value)
Reduce Perfusion	2 (33.3)	25 (89.3)	0.002
Heteroechogenicity	1 (16.7)	15 (53.6)	0.046
Hypoechogenicity (Homogenous)	1 (16.7)	8 (28.6)	0.046
Increase Size	3 (50.0)	12 (42.9)	0.948
Decrease Size	2 (33.3)	11 (39.3)	0.948

**Table 4:** Sonographic findings of patients presenting with TT and surgical outcome

Features	TT group, N= 34 N (%)	Non-TT group, N=19 N (%)	Chi-square (P-value)
Reduce Perfusion	27 (79.4)	4 (21.1)	<0.001
Heteroechogenicity	16 (47.1)	4 (21.1)	0.003
Hypoechogenicity (Homogenous)	9 (26.5)	1 (5.3)	0.003
Incresease Size	15 (44.1)	6 (31.6)	0.024
Decrease Size	13 (38.2)	3 (15.8)	0.024

The analysis of admission time to surgery was done by using Mann-Whitney U-test and it was noted that the time duration from admission to surgery has no significant difference between the patient with and without ultrasound evaluation (P=0.078). Furthermore, it also shows no significant difference between the time of admission to surgery and the outcome. (P=0.521). Patients who did ultrasound had a higher rate of orchidectomy compared to those that underwent orchidopexy (n=32/36, OR:5.82, P=0.008). This may be due to the high frequency of patients who had an ultrasound done presented with a pain duration >24 Hours (43/53, 81.1%).

## Discussion

Our study showed that in 88 patients with acute scrotal pain, only 62.5% had testicular torsion while the remaining underwent unnecessary surgical exploration. The rate of negative surgical exploration based on clinical judgment alone was 45.5%. Many researchers suggest that clinical examination alone would reduce the negative exploration rate by 55%. However, the negative surgical exploration rate is reduced by 59% by combining clinical assessment with ultrasound findings [10]. Thus, it is crucial to obtain a good medical history, appropriate clinical evaluation and ultrasound of scrotum to determine the most probable diagnosis. In our study when the clinical examination was combined with the US the negative exploration rate was reduced by 10%.

Testicular torsion requires prompt diagnosis and it is a race against time in achieving testicular viability. With each passing hour, the blood flow to the testis diminishes thereby leading to higher chances of orchidectomy. Surgical exploration is done to ensure the definite cause in these patients. However certain conditions presenting as scrotal pain do not require surgical exploration and can be treated medically. For example, epididymo-orchitis closely resembles testicular torsion and is discovered in 19% of the patients who underwent surgical exploration in our study.

There is typically a four to eight-hour window before significant ischemic damage occurs, manifested by morphologic changes in testicular histopathology and deleterious effects on spermatogenesis [11]. It is important to note that imagining should not cause delay and when in doubt exploration of the scrotum should be done. Reported testicular salvage rates are 90% to 100% if surgical exploration is performed within six hours of symptom onset, decreases to 50% if symptoms are present for more than 12 hours, and are typically less than 10% if symptom duration is 24 hours or more [1, 12]. In our study time since pain and performing the US did not impact the outcome as our patients presented late. It was noted that the patients who had the US had a higher rate of orchidectomy and this was attributed to performing the US in patients with pain >24 hours. Based on the time of admission to surgery, patients with testicular torsion who underwent orchidopexy had an average time of 544 mins, whereas those with testicular torsion with Orchidectomy had an average time of 443.76 mins (p=0.052). Time since admission was not significantly different, indicating that the length of time from admission to surgery may not be a significant factor in determining the viability of testis. Furthermore, a comparison of average time in patients with and without an ultrasound result, which is 618.06 minutes and 402.45 minutes respectively is shown to be insignificant (P=0.078). Thus, we can conclude that performing ultrasound did not have an impact on the outcome.

Ultrasonography (US) is a common imaging modality in patients with acute scrotal pain. The normal testes are homogeneous oval-shaped structures with medium reflectivity on B-mode ultrasound, their size varying with age and stage of sexual development. In the adult male, they measure about 4cmx3cmx2.5cm [13]. In the early phases of torsion (1-3 hours), testicular echogenicity appears normal. A complete or a partial hypoechoic pattern maybe an early sonographic sign. With progression, enlargement of the affected testis and increased or heterogeneous echogenicity are common findings [14]. Color Doppler ultrasonography (CDUS) evaluates the size, shape, echogenicity, and perfusion of both testicles. Color Doppler imaging of testicular torsion demonstrates a relative decrease or absence of blood flow within the affected testicle [9]. If blood flow is absent on Doppler imaging and history and clinical findings are consistent with torsion, then immediate surgical exploration is required. In the case of inflamed epididymis and testis, it shows increased blood flow. On CDUS arterial and venous flow may be absent or may show a reduced or reversed diastolic arterial flow. This finding can be indicative of early or partial torsion, given that the arterial system requires a higher pressure to occlude flow than does the venous system [15]. Meticulous sonographic evaluation of the spermatic cord and the opposite testis are important in the diagnosing torsion. With an improvement in ultrasound and Doppler capabilities, many studies now report sensitivities and specificities of sonography for testicular torsion between 89 and 100% [16].

In our study Ultrasound evaluation of TT showed a sensitivity and specificity of 88.24% and 68.40% respectively. The positive predictive value was 83.33% and the negative predictive value was 76.47%. Thus, we conclude that ultrasound can be a good tool and when positive findings are present the patient will require orchidectomy. We have noted that ultrasound findings of heteroechogenicity or homogenous hypoechogenicity are more often seen in TT rather than in patients diagnosed with other pathologies. CDUS showed reduced perfusion of the affected testis is more associated in the TT group. It is important to note that out of 34 patients diagnosed with TT, 27 had reduced perfusion on US findings, 16 showed heteroechogenicity whereas the US showed 9 patients with hypo-echogenicity. Therefore, it is reasonable to conclude that patients with US finding of hetero-echogenicity are at higher risk of having TT as compared to hypoechogenicity. Another important finding is that a significant number of patients underwent orchidectomy had abnormal US findings which include changes in echogenicity, testicular size, and decreased perfusion. Age is important in the diagnosis of TT. We did not encounter neonatal cases in our study, and the mean age of patients presenting with testicular pain was 18 years. Our study showed that age is not a good indicator to diagnose testicular torsion as there is no significant difference in the mean age of those with testicular torsion or a different pathology. Furthermore, the mean age of those patients who underwent orchidectomy and orchidopexy was approximately 17 and 21 years old respectively as indicated by the p-value of (0.046).

Testicular torsion is clinically correlated with symptoms such as nausea, vomiting, fever, scrotal swelling and urinary symptoms [17]. Scrotal swelling had a significant correlation with testicular torsion in our study and there is no statistically significant correlation between the presenting symptoms (nausea, vomiting, fever, dysuria) and testicular torsion; most patients that presented with scrotal pain have an associated swelling. Despite this, swelling is not a good indicator to differentiate between testicular torsion and another testicular pathology as the negative predictive value is low 53.12%. However, other studies have shown a pathological cremasteric reflex on the ipsilateral side and high riding testis to be more associated with testicular torsion [7]. We recommend that patients with strong clinical suspicion of testicular torsion should undergo surgical exploration whereas those with an uncertain diagnosis should be sent for an ultrasound.

**Limitations:** the study consists of a small sample size of only 88 patients with acute scrotal pain. With a larger sample size, our data may have more statistical power and be better for extrapolation to the general population. Apart from that, the data collected is based on one hospital population only. Furthermore, some patients´ sonographic findings were incomplete due to the unavailability of data in the patient´s records. Since the ultrasound image was reviewed by a different radiologist at different times, the definition of heterogeneous echotexture may be different in comparison to that used in previously published studies as there may be some variation. Thus, it would be ideal to standardize the definition for “heterogeneous echotexture” for future studies for better comparisons

## Conclusion

To determine the most probable diagnosis of Testicular Torsion (TT), it is crucial to obtain a good medical history, appropriate clinical evaluation and performing an ultrasound of the scrotum. Scrotal swelling has a significant correlation with testicular torsion. Despite this, it is not a good indicator to differentiate between testicular torsion and other pathology. Reduced testicular perfusion, hypo-/heterogeneous echotexture has shown to be a good imaging indicator in our study. Duration of pain to admission of more than 6 hours, reduced testicular perfusion, hypo-/heterogenous echotexture, and change in testicular size are good indicators for testicular non-viability. US when paired with clinical judgment, could reduce the rate of negative exploration.

### What is known about this topic

- Testicular torsion present with acute scrotal pain and is a surgical emergency;
- Early diagnosis and timely intervention are important inn salvaging the testis;
- Time lost in investigations can delay treatment.

### What this study adds

- It is important to obtain a good medical history, appropriate clinical evaluation and performing an ultrasound of the scrotum;
- Reduced testicular perfusion, hypo-/heterogeneous echotexture on ultrasonography has shown to be a good imaging indicator in our study;
- Ultrasonography when paired with clinical judgment, could reduce the rate of negative exploration.

## Competing interests

The authors declare no competing interests.
